# Endoplasmic reticulum–mitochondria contact sites in benign prostatic hyperplasia: evidence integration and mechanistic hypotheses

**DOI:** 10.3389/fimmu.2026.1945942

**Published:** 2026-10-07

**Authors:** Lei Liu, Minhao Fu, Zhenting Wang

**Affiliations:** Haikou Research Institute of Xiangya School of Medicine, Central South University, Haikou, China

**Keywords:** ageing, benign prostatic hyperplasia, endoplasmic reticulum stress, inflammation, mitochondria-associated membranes, mitochondrial dysfunction, oxidative stress

## Abstract

Benign prostatic hyperplasia (BPH) is a common chronic and progressive disease in ageing men. Its pathobiology involves androgen-signaling remodeling, chronic inflammation, oxidative stress, metabolic disturbance, and dysregulated cell fate control; however, the organelle-level communication that connects these processes remains insufficiently defined. Endoplasmic reticulum (ER)–mitochondria contact sites, commonly referred to as mitochondria-associated membranes (MAMs), are dynamic membrane contact platforms that coordinate ER stress with mitochondrial homeostasis. Through the regulation of Ca²^+^ transfer, lipid metabolism, mitochondrial dynamics, autophagy, inflammatory signaling, and cell death, MAMs may provide a structural and functional interface linking several pathological features of BPH. In this review, we integrate evidence from BPH studies, including GRP78 upregulation, mitochondrial reactive oxygen species (ROS) accumulation, NLRP3 inflammasome activation, DRP1-associated abnormalities in mitochondrial dynamics, and resistance to apoptosis. On this basis, we discuss the mechanistic hypothesis that MAM remodeling may contribute to the chronic progression of BPH. Current evidence does not support defining MAMs as direct pathogenic drivers of BPH. Rather, positioning MAMs as an organelle-communication framework that links ER stress, mitochondrial injury, and tissue hyperplasia may help explain the pathological heterogeneity of BPH and identify candidate targets for future investigation.

## 1 Introduction

Benign prostatic hyperplasia (BPH) is a common chronic and progressive urological disease in ageing men, and its prevalence increases substantially with age. The clinical burden of BPH extends beyond prostate enlargement and is mainly reflected in the long-term effects of lower urinary tract symptoms (LUTS) on voiding function, sleep quality, and overall quality of life (1, 2). α1-Adrenoceptor blockers, 5α-reductase inhibitors, and surgical interventions remain the principal therapeutic strategies. However, these approaches mainly provide symptomatic relief or delay progression in selected patient groups. Because BPH is clinically heterogeneous and evolves over many years, variable treatment responses, persistent progression, and the need for alternative therapeutic options remain major challenges in clinical management (2, 3). BPH should therefore be understood not simply as glandular enlargement, but as a chronic ageing-related disease associated with alterations in the local tissue microenvironment and disruption of cellular homeostasis.

The development and progression of BPH have traditionally been interpreted through androgen receptor (AR) signaling, dihydrotestosterone (DHT) production, and chronic inflammation. This framework remains essential for understanding the disease. Recent evidence indicates that inflammatory processes may enhance AR activity in basal prostate stem cells through mediators such as IL-1RA, thereby promoting proliferation, luminal differentiation, and the emergence of a hyperplastic phenotype (4). Nevertheless, circulating androgen levels alone do not adequately explain the heterogeneity of clinical manifestations and pathological changes in BPH. A *post hoc* analysis of the REDUCE study found no significant association between baseline serum testosterone or DHT levels and the occurrence or progression of LUTS (5). These observations suggest that local steroid metabolism within the prostate, inflammation-associated remodeling of signaling networks, and stress-related metabolic adaptation may jointly shape BPH phenotypes (4, 6).

This complexity is also reflected in pathological evidence involving organelle stress and impaired mitochondrial homeostasis. Fu et al. reported that the endoplasmic reticulum (ER) chaperone GRP78 is upregulated in BPH tissues. In experimental models, GRP78 knockdown inhibited cell proliferation and induced apoptosis, accompanied by changes related to oxidative stress and epithelial-to-mesenchymal phenotypic transition (7). At the mitochondrial level, MitoQ has been shown to reduce mitochondrial reactive oxygen species (ROS) generation and inhibit prostate epithelial cell proliferation by suppressing AR–NLRP3-related signaling. Korean red ginseng may also modulate DRP1-mediated mitochondrial fission (8, 9). Taken together, these findings suggest that oxidative stress, ER stress, mitochondrial dysfunction, inflammatory amplification, and resistance to cell death may not be merely isolated downstream events; rather, they may converge at the level of organelle communication.

ER–mitochondria contact sites, commonly referred to as mitochondria-associated membranes (MAMs), provide a structural and functional framework for integrating these pathological alterations. MAMs are dynamic contact regions formed between the ER and mitochondria. They participate in Ca²^+^ exchange, lipid transfer, energy metabolism, mitochondrial dynamics, cell fate regulation, and the amplification of inflammatory signaling (10–12). From the perspective of organelle communication, GRP78 upregulation, mitochondrial ROS accumulation, NLRP3 inflammasome activation, DRP1-mediated imbalance in mitochondrial dynamics, and the enhanced anti-apoptotic state reported in BPH all overlap with biological processes regulated by MAMs. On this basis, the present review integrates the relatively scattered evidence on organelle stress in BPH and proposes a testable mechanistic hypothesis centered on MAMs. At present, MAMs should not be described as established pathogenic factors in BPH. A more cautious interpretation is that they may serve as an organelle communication platform linking ER stress, mitochondrial injury, oxidative stress, inflammatory amplification, and resistance to cell death.

## 2 Structural and functional basis of MAMs

### 2.1 Structural characteristics of MAMs

ER–mitochondria contact sites are specialized membrane contact regions in which the two organelles are typically separated by a nanoscale distance of approximately 10–30 nm. These domains are commonly referred to as mitochondria-associated ER membranes. Rather than serving as static adhesive structures, MAMs are dynamic interfaces that can be remodeled according to metabolic status, Ca²^+^ load, and stress intensity (13, 14). Their stability is maintained by multiple tethering proteins and functional complexes. Variations in contact distance, local protein composition, and the membrane microenvironment may influence the efficiency of spatial coupling, material exchange, and signal transduction between the ER and mitochondria.

Among MAM-associated molecules, the IP3R–GRP75–VDAC1 complex is a canonical axis linking ER Ca²^+^ release to mitochondrial Ca²^+^ uptake. By generating Ca²^+^-enriched microdomains at contact sites, this complex supports efficient local coupling between ER Ca²^+^ release and mitochondrial Ca²^+^ entry (15, 16). MFN2 contributes to interorganelle communication and mitochondrial fusion, but its precise role in ER–mitochondria contact regulation remains debated. Some studies support a tethering role, in which MFN2 loss reduces close contacts, whereas Filadi et al. reported that MFN2 ablation increases structural and functional ER–mitochondria coupling, consistent with a spacer or negative-regulator role (17, 18). MFN2 should therefore be considered a context-dependent regulator of ER–mitochondria contact organization rather than a unidirectional tether. Other MAM-associated regulators have complementary functions: PACS2 helps organize membrane architecture at contact sites; Sig-1R functions as a local chaperone involved in stress responses and Ca²^+^ homeostasis; and PERK may contribute to contact-site remodeling under stress, enabling ER stress signals to be transmitted toward mitochondria (19, 20). Thus, MAMs are not only structural scaffolds but also platforms for signal integration, providing an organelle-level framework for interpreting the reciprocal amplification of Ca²^+^ imbalance, ER stress, and mitochondrial injury (**Figure 1**).

**Figure 1:**
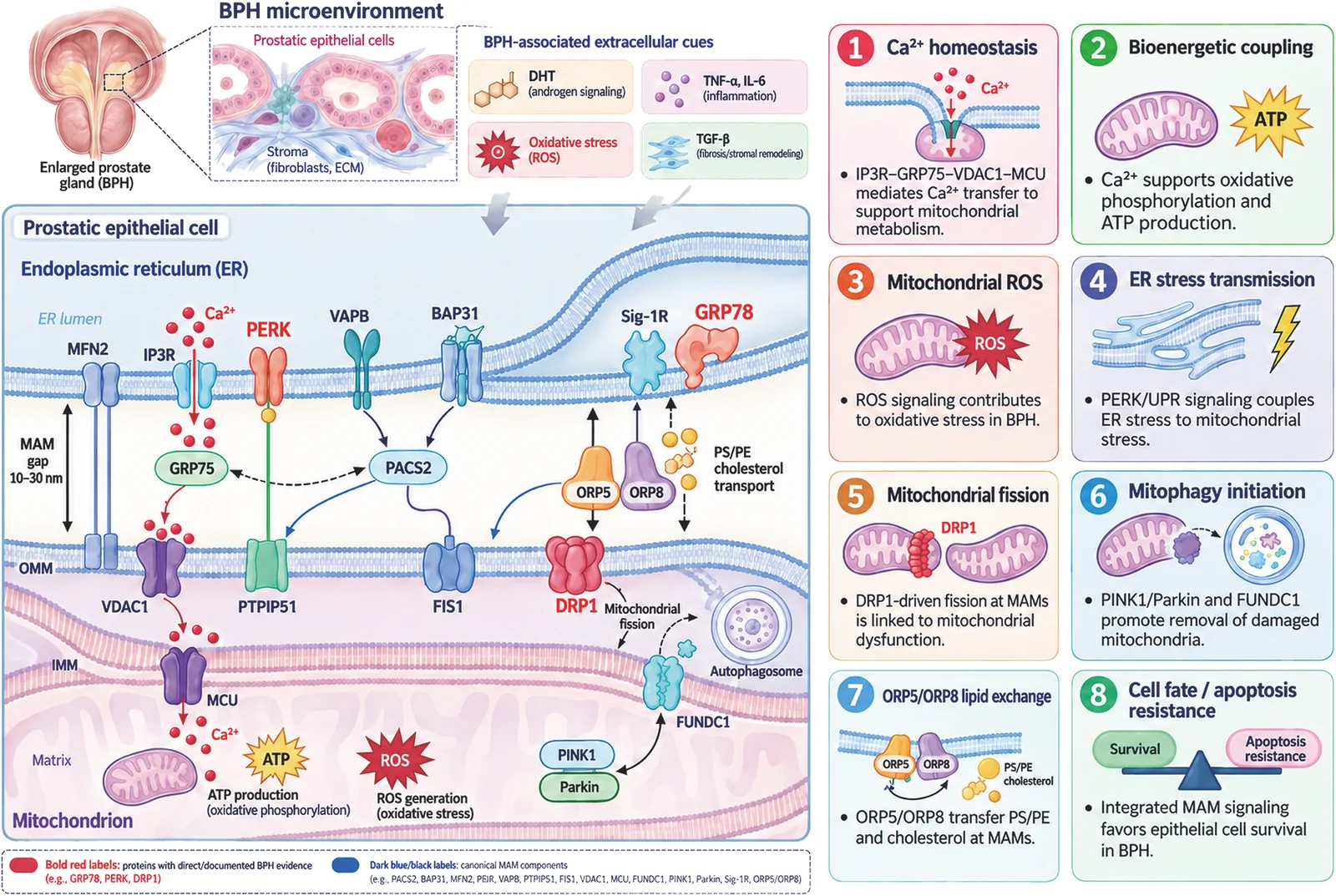
BPH-oriented schematic of endoplasmic reticulum–mitochondria contact sites (MAMs) in a prostatic epithelial cell. The upper panel places ER–mitochondria communication within a BPH-associated microenvironment characterized by androgenic stimulation, inflammatory cytokines, oxidative stress, and stromal remodeling. The central MAM interface depicts major structural and functional components, including the IP3R–GRP75–VDAC1–MCU Ca²^+^-transfer axis, MFN2, VAPB–PTPIP51, BAP31–FIS1, PACS2, Sig-1R/GRP78, PERK, DRP1, FUNDC1, and PINK1/Parkin. Proteins with BPH-related evidence discussed in this review, including GRP78, PERK, and DRP1, are highlighted in bold red, whereas canonical MAM components with limited BPH-specific validation are shown in dark blue or black. ORP5 and ORP8 are incorporated into the lipid-transfer module to illustrate molecularly defined phospholipid exchange at ER–mitochondria contact sites, together with broader MAM-associated lipid remodeling. The functional panels summarize Ca²^+^ homeostasis, bioenergetic coupling, mitochondrial ROS generation, ER-stress transmission, mitochondrial fission, mitophagy initiation, lipid exchange and membrane remodeling, and cell-fate regulation. The BPH-associated microenvironmental cues shown in the figure provide disease-specific context and should not be interpreted as direct evidence that these factors cause MAM remodeling in BPH.

### 2.2 Core functions of MAMs

#### 2.2.1 Ca²^+^ transfer and regulation of mitochondrial bioenergetics

MAM-mediated Ca²^+^ transfer from the ER to mitochondria is essential for maintaining mitochondrial bioenergetic homeostasis. Through the IP3R–GRP75–VDAC1 complex, Ca²^+^ released from the ER forms high-concentration microdomains at contact sites and can be rapidly taken up by adjacent mitochondria. After entering the mitochondrial matrix, Ca²^+^ activates key enzymes of the tricarboxylic acid cycle, increases NADH production and oxidative phosphorylation, and thereby supports ATP generation (21, 22). This process helps meet the energy requirements for protein folding, ionic homeostasis, and adaptation to cellular stress.

Maintenance of this Ca²^+^ flux also depends on replenishment of the ER Ca²^+^ store. The sarco/endoplasmic reticulum Ca²^+^-ATPase (SERCA), particularly the widely expressed SERCA2b isoform, uses ATP to transport cytosolic Ca²^+^ back into the ER lumen, thereby restoring Ca²^+^ available for subsequent release through channels such as IP3Rs and RyRs. Local regulation of SERCA2b can therefore influence ER–mitochondria Ca²^+^ transfer and mitochondrial bioenergetics (23). Pharmacologically, thapsigargin is a well-established SERCA inhibitor that blocks ER Ca²^+^ reuptake and progressively depletes ER Ca²^+^ stores, making it a useful experimental tool for studying ER Ca²^+^ homeostasis and ER–mitochondria Ca²^+^ signaling (24).

MAM-mediated Ca²^+^ flux is highly dependent on both dose and duration. Moderate Ca²^+^ input sustains mitochondrial metabolism, whereas persistent or excessive Ca²^+^ transfer may increase mitochondrial Ca²^+^ loading, reduce membrane potential, enhance ROS generation, and promote opening of the mitochondrial permeability transition pore (mPTP) (25). Conversely, insufficient Ca²^+^ input may weaken mitochondrial metabolic activity and reduce ATP production efficiency (26). Therefore, MAM function should not be interpreted as inherently protective or detrimental. Its biological outcome depends on contact strength, Ca²^+^ flux, and the specific stress context. In a chronic progressive disease such as BPH, this context-dependent feature may help explain the coexistence of restricted energy metabolism, increased oxidative burden, and abnormal stress adaptation.

#### 2.2.2 Mitochondrial dynamics and quality control

MAMs also participate in remodeling and quality control of the mitochondrial network. Mitochondrial fission is not confined to mitochondria themselves; it often begins at ER–mitochondria contact regions. These regions may serve as pre-fission positioning sites, where the ER locally wraps around mitochondria and recruits fission-related molecules such as DRP1, helping define the site of mitochondrial division (16, 27, 28). Under sustained metabolic stress, contact-site-associated fission signaling may be amplified, shifting the mitochondrial network from a relatively continuous state toward fragmentation. In the short term, this shift may represent an adaptive stress response. If sustained, however, it may interact with oxidative stress and further disrupt cellular homeostasis.

At the level of quality control, MAMs contribute to the recognition and segregation of damaged mitochondria and to the initiation of mitophagy. Both the PINK1–Parkin pathway and FUNDC1-related mechanisms are closely linked to the structural integrity of contact sites, and their dysfunction may impair the clearance of damaged mitochondria (29). In various disease models, MAM dysfunction is often accompanied by reduced mitophagy, accumulation of damaged mitochondria, and sustained ROS release. Given the age-related nature of BPH, impaired mitochondrial quality control during ageing and chronic stress may allow damaged mitochondria to persist, potentially linking chronic oxidative stress, inflammatory amplification, and tissue remodeling (27, 30, 31). Current evidence remains insufficient to define this process as an independent pathogenic pathway in BPH. Nevertheless, its overlap with recognized BPH pathological features warrants further validation.

#### 2.2.3 ER stress, lipid exchange, and cell fate regulation

MAMs provide a spatial interface through which ER stress can be transmitted to mitochondria. This relationship extends beyond the colocalization of stress-related proteins. PERK is enriched at ER–mitochondria contact sites and contributes to the maintenance of ER–mitochondria juxtaposition. Under ROS-associated ER stress, this contact-site function can facilitate stress-signal propagation toward mitochondria and influence apoptotic sensitivity (32). IRE1α also has a non-canonical role at MAMs. It can act as a structural scaffold that helps organize IP3R distribution at contact sites and thereby regulates mitochondrial Ca²^+^ uptake and bioenergetics (33). Sig-1R provides another stress-sensitive mechanism. Under resting conditions, Sig-1R associates with GRP78 at MAMs. ER Ca²^+^ depletion promotes dissociation of this complex and facilitates Sig-1R-dependent stabilization of IP3R3, thereby sustaining Ca²^+^ transfer to mitochondria (34). Through these mechanisms, ER stress can influence mitochondrial Ca²^+^ loading, ROS production, energy metabolism, and cell-fate signaling. The biological outcome remains stress dependent: adaptive signaling may support survival during moderate chronic stress, whereas persistent or severe stress may increase mitochondrial dysfunction and cell-death signaling.

Beyond Ca²^+^ signaling, lipid exchange is another established function of ER–mitochondria contact sites. ORP5/ORP8 mediate non-vesicular phosphatidylserine transfer at these contacts through PTPIP51 and the MIB/MICOS machinery, whereas VPS13D can bridge the ER and mitochondria through VAP- and Miro-dependent interactions (35–37). ATAD3-dependent MAM formation has also been linked to mitochondrial cholesterol delivery in steroidogenic cells (38). However, direct evidence for dysregulation of these pathways in BPH is currently lacking. We therefore consider MAM-associated lipid handling as background contact-site biology rather than an established BPH-specific pathogenic mechanism.

Recent mechanistic studies indicate that ER–mitochondria contact remodeling can modulate ferroptosis susceptibility in non-BPH systems. Pharmacological targeting of the MAM-resident chaperone Sig-1R or genetic disruption of MAMs impaired ER-to-mitochondria Ca²^+^ transfer, reduced mitochondrial ROS production, and increased the sequestration of PUFAs in triacylglycerols, thereby suppressing ferroptosis (39). A subsequent study identified ER–mitochondria contact sites as early hotspots of phospholipid peroxidation: contact-site untethering reduced phospholipid peroxidation and ferroptosis, whereas contact stabilization enhanced both (40). These findings provide mechanistic context for a possible relationship among MAM remodeling, Ca²^+^/ROS coupling, phospholipid peroxidation, and ferroptosis. However, these studies were conducted outside BPH models, and a MAM-dependent ferroptosis pathway has not been demonstrated in BPH. Accordingly, ferroptosis-related mediators are considered here as exploratory mechanistic context rather than as components of an established BPH pathway.

At ER–mitochondria contact sites, ER stress, mitochondrial dysfunction, lipid metabolic remodeling, and cell fate regulation may be functionally interconnected rather than occurring as isolated events. Based on the structural and functional features described above, this review further summarizes MAM-associated molecules and functional modules that may be involved in BPH pathology. It should be emphasized that some molecules listed in **Table 1** have BPH-related evidence, whereas others are mainly supported by studies in other disease models or by research on organelle communication. Their significance should therefore be interpreted as mechanistic clues and priorities for future validation, rather than as established pathogenic targets in BPH.

**Table 1:** MAM-associated molecules and functional modules: potential roles in BPH pathophysiology and priorities for validation.

MAM-associated molecule/module	BPH/prostate-context evidence	Evidence boundary	Priority for BPH-specific validation
IP3R–GRP75–VDAC1–MCU axis	Direct BPH-specific evidence for remodeling of the complete axis is currently limited.	The ER-to-mitochondria Ca²^+^-transfer function is well established in general MAM biology (97), but altered contact-site Ca²^+^ flux has not been directly demonstrated in BPH.	PLA/co-IP, mitochondrial Ca²^+^ imaging, ΔΨm, ATP/OCR and mtROS after MAM-node perturbation.
MFN2	No direct BPH-specific evidence of MFN2-dependent MAM remodeling has been established.	MFN2 regulates both ER–mitochondria communication and mitochondrial fusion (18); therefore, mitochondrial phenotypes alone cannot be attributed specifically to MAM remodeling.	MFN2 IHC/IF, TEM contact metrics and gain/loss-of-function studies in BPH-1, WPMY-1 or organoids.
PACS2	No direct BPH-specific evidence identified.	Current support is derived mainly from general MAM and ER–mitochondria communication studies (98, 99).	Spatial localization, loss-of-function and contact-structure analysis in prostate models.
Sig-1R	Prostate-context molecular evidence is available, but direct BPH-specific MAM evidence remains limited.	Sig-1R participates in MAM-associated stress and Ca²^+^ regulation (100, 101), but its contribution to physical MAM remodeling in BPH is unproven.	Expression profiling, MAM localization and pharmacological perturbation with ER-stress and functional readouts.
GRP78	GRP78 upregulation has been reported in BPH tissues (7).	GRP78 is strong evidence of ER-stress/UPR activity, but its upregulation alone does not demonstrate physical MAM remodeling.	Tissue IHC/WB, MAM localization, siRNA and proliferation/apoptosis assays.
PERK	PERK-related UPR abnormalities are relevant to the chronic ER-stress phenotype of BPH.	Its role as a MAM-associated stress platform is supported mechanistically (32), but BPH-specific contact-site localization remains unproven.	Assess p-PERK, MAM localization, UPR output, mtROS and apoptosis after PERK perturbation.
DRP1	BPH-related abnormalities in mitochondrial fission provide comparatively stronger disease-context support.	DRP1-mediated fission can originate at ER-marked mitochondrial sites (28), but altered fission alone does not demonstrate MAM remodeling.	DRP1 phosphorylation, fission index, mitochondrial morphology, MAM structure, ROS and proliferation.
PINK1/Parkin and FUNDC1	PINK1/Parkin-related abnormalities have BPH relevance, whereas FUNDC1 remains more exploratory.	These proteins participate in mitochondrial quality control and MAM-associated mitophagy (102), but their MAM dependence in BPH remains unproven.	Mitophagy flux, Parkin recruitment, FUNDC1, LC3/p62/TOM20 and mitochondrial-clearance assays.
Bcl-2 family proteins	Bcl-2/Bax imbalance and reduced apoptotic activity have been reported in BPH.	These findings support apoptosis resistance but do not establish that the phenotype is MAM-dependent (103).	Bcl-2/Bax, cleaved caspase-3, TUNEL and rescue experiments combined with MAM perturbation.
NLRP3 inflammasome/TXNIP–NLRP3 interface	NLRP3-related inflammatory activation is supported in BPH-related experimental contexts.	The mtROS–NLRP3 relationship is biologically plausible, but direct MAM dependence remains unclear (12, 70).	mtROS, NLRP3/ASC, cleaved caspase-1, IL-1β/IL-18 and ER–mitochondria proximity.
ORP5/ORP8	No direct BPH-specific evidence identified.	Evidence currently derives from general MAM lipid-transfer biology, including ER-to-mitochondria phosphatidylserine transfer (37).	MAM localization, ORP5/8–PTPIP51 proximity, PS-to-PE flux, lipidomics and lipid ROS.
VPS13D	No BPH-specific evidence identified	Current evidence supports a VAP/Miro-dependent ER–mitochondria bridging function in other cellular systems (36).	VPS13D localization, VAP/Miro dependence, MAM proximity, lipidomics and mitochondrial morphology.
ATAD3	No direct BPH validation identified.	Evidence for MAM formation and mitochondrial cholesterol delivery is derived mainly from steroidogenic systems (38).	ATAD3 localization, MAM structure, cholesterol transfer and androgen/oxidative-stress responses in prostate cells.
ACSL4–ALOX12/15–GPX4	Current BPH evidence is indirect and hypothesis-generating.	These molecules regulate lipid-peroxidation and ferroptosis-related processes (104–107), but a MAM-dependent ferroptosis pathway has not been established in BPH.	Combine ACSL4/ALOX/GPX4 measurements with lipid ROS, 4-HNE/MDA, ferroptosis rescue and direct MAM assays.

Among the nodes discussed above, Ca²^+^ transfer, mitochondrial dynamics, ER stress, mitochondrial quality control, inflammatory amplification, and lipid peroxidation substantially overlap with oxidative stress, chronic inflammation, an anti-apoptotic state, and tissue remodeling previously reported in BPH. The next section examines which pathological alterations reported in BPH may be related to MAM-associated processes, while distinguishing available evidence from mechanistic hypotheses that still require validation.

## 3 Pathological evidence in BPH potentially linked to MAMs

### 3.1 Oxidative stress, inflammation, and ER stress

Oxidative stress and chronic inflammation are recurrent pathological features of BPH and provide one of the most relevant disease contexts for interpreting MAM-related functions. Evidence from animal models and clinical studies indicates that hyperplastic prostate tissues often show increased lipid peroxidation, ROS accumulation, and reduced antioxidant enzyme activity, together with activation of TNF-α, IL-1β, IL-6, and NF-κB signaling (41, 42). Metabolic disorders and elevated uric acid may further aggravate local oxidative and inflammatory stress and activate pro-survival pathways, including PI3K–Akt–mTOR and NF-κB, thereby contributing to cell proliferation and an anti-apoptotic phenotype (43).

The inflammatory microenvironment of BPH also includes an active immune-cell compartment rather than cytokine signaling alone. Recent single-cell analysis identified an age-associated CD8^+^ T-cell subset characterized by high GZMK and low GZMB expression in aged human prostates. Its abundance was positively associated with IPSS, linking immune ageing with symptomatic BPH. Granzyme K stimulation also increased SASP-associated cytokine production in BPH patient-derived prostate fibroblasts, suggesting a potential T-cell–stromal inflammatory circuit (44). These findings support bidirectional communication among immune cells, senescent or stressed stromal cells, and the prostate epithelium within the chronic inflammatory microenvironment.

ER stress and activation of the unfolded protein response (UPR) provide another mechanistic link between BPH-related pathological phenotypes and MAM biology. GRP78 has been reported to be upregulated in BPH tissues, and its expression is closely associated with prostate cell proliferation, oxidative stress, and epithelial–mesenchymal transition (EMT)-related changes (7). Experimental studies in BPH models further suggest that ER stress-related molecules, including PERK, ATF6, and CHOP, participate in the regulation of autophagy, apoptosis, and cell survival (45, 46). However, persistent ER stress in BPH does not appear to result in extensive cell death. Instead, it often coexists with anti-apoptotic features, such as increased Bcl-2 expression, reduced Bax expression, and attenuated caspase-3 activity (47, 48). This pattern suggests that prostate cells may acquire adaptive survival responses under chronic stress conditions.

### 3.2 Mitochondrial dysfunction and impaired quality control

Mitochondrial dysfunction and impaired quality control provide important indirect evidence for the potential involvement of MAMs in BPH pathology. Ageing-related studies have shown impaired mitochondrial complex I function in BPH tissues, accompanied by downregulated expression of mitochondria-associated proteins, including NDUFS3, VDAC1, VDAC2, and PINK1. These alterations are associated with compromised oxidative phosphorylation, increased collagen deposition, and upregulation of fibrosis-related genes (49). Mitochondrial injury may chronically increase ROS burden, promote inflammatory and fibrotic responses, and contribute to low-grade tissue injury and dysregulated repair. Available evidence also suggests that DRP1 and Parkin-mediated mitochondrial dynamics and mitophagy may be involved in BPH-associated pathological phenotypes (50). Although these findings do not directly demonstrate changes in MAM contact architecture, MAMs are recognized spatial platforms for mitochondrial fission, autophagy initiation, and organelle quality control. They may therefore offer a plausible mechanistic framework for linking damaged mitochondrial accumulation, sustained ROS release, and prostate tissue remodeling.

Importantly, the BPH-associated readouts summarized above should be interpreted as markers of organelle stress and altered functional output rather than as direct evidence of remodeling of the physical MAM scaffold. GRP78 upregulation primarily reflects ER stress and UPR activity, whereas Complex I dysfunction and alterations involving NDUFS3, VDAC1/2, PINK1, DRP1, or Parkin predominantly indicate abnormalities in mitochondrial bioenergetics, dynamics, or quality control (7, 49, 50). None of these readouts, in isolation, establishes altered ER–mitochondria contact distance, contact length or frequency, or contact-site protein proximity. Therefore, direct structural measurements and MAM-dependent functional assays, as outlined in **Table 2**, are required before physical MAM remodeling can be concluded in BPH.

**Table 2:** Experimental models, validation approaches, and key criteria for evaluating the MAM–BPH hypothesis.

Research layer	Recommended model/sample	Methodologiwecal approach	Key readouts and decision criteria
Human tissue structural validation	TURP-derived BPH specimens, transition-zone tissues and age-matched non-hyperplastic controls when available.	TEM/3D-EM, super-resolution microscopy, IF and PLA (115).	Contact length/frequency/ratio, ER–mitochondria distance and MAM-protein colocalization; direct support requires reproducible contact remodeling in BPH tissues.
MAM protein and cell-type mapping	BPH tissues with inflammatory, fibrotic or hyperplastic phenotypes; epithelial, stromal, smooth muscle, immune and endothelial compartments (44, 116).	IHC, multiplex IF, PLA, proteomics, scRNA-seq and spatial transcriptomics.	MFN2, GRP75, VDAC1, GRP78 PERK, PACS2, Sig-1R, FUNDC1, DRP1 and PINK1/Parkin localization; support increases with cell-type specificity and pathology correlation.
Functional Ca²^+^ and mitochondrial validation	BPH-1, WPMY-1, primary prostate cells and inflammatory/androgen-stimulated models.	MAM-node knockdown/overexpression, pharmacological modulation, ER-stress induction, DHT/TNF-α/IL-1β stimulation, Ca²^+^ imaging and Seahorse OCR (116, 117).	Cytosolic/mitochondrial Ca²^+^, ΔΨm, OCR, ATP, mPTP, mtROS and proliferation/apoptosis; MAM involvement requires phenotype changes after MAM perturbation.
Inflammation and epithelial–stromal interaction models	BPH-1/WPMY-1 co-culture, conditioned-medium systems, primary epithelial–stromal co-cultures and cytokine-stimulated models.	Co-culture, cytokine stimulation, MAM manipulation, inflammasome assays, ELISA, western blot and IF.	IL-6, TNF-α, IL-1β/IL-18, NF-κB, NLRP3/ASC, cleaved caspase-1, TGF-β/Smad, EMT and collagen markers; support requires altered inflammatory remodeling (4).
Mitochondrial dynamics and quality-control validation	Gene-edited BPH-1/WPMY-1 cells, primary cells, co-cultures and stress-induced mitochondrial-damage models.	siRNA/shRNA, CRISPR, overexpression of MFN2, GRP75, FUNDC1, DRP1, PERK or Sig-1R; mitophagy reporters and live-cell imaging (8).	DRP1 phosphorylation, fragmentation, PINK1/Parkin recruitment, FUNDC1, LC3/p62/TOM20, mitophagy flux, clearance, ROS and apoptosis; causality requires altered damage accumulation and growth/survival.
Patient-derived organoid and tissue-engineering models	Human/BPH prostate organoids, stromal/immune-incorporated organoids and engineered prostate microenvironments.	3D culture, inflammatory/androgen stimulation, MAM-node perturbation, genetically encoded split-GFP-based proximity biosensors (e.g., SPLICS) for dynamic ER–mitochondria proximity sensing, live imaging, drug-response testing and molecular profiling (81, 118).	Organoid growth, branching, differentiation, ROS, ER stress, mitophagy, apoptosis, EMT-like change and drug response; bridges cell culture with patient-level relevance.
Animal and preclinical validation	Testosterone-induced, ageing-related, prolactin-related and metabolic syndrome-related BPH models.	Pharmacological/genetic modulation when feasible, including conditional knockout models for causal validation (e.g., Mfn2^^fl^/fl crossed with a prostate epithelial Cre driver such as PB-Cre4), histology, IF/IHC, TEM, urinary function and molecular profiling (18, 118, 119).	Prostate weight, epithelial thickness, stromal ratio, fibrosis, inflammation, urinary parameters, MAM structure, ER stress, mtROS and mitophagy; relevance requires phenotype improvement plus MAM change.
Clinical association and translational stratification	Prospective BPH cohorts with tissue, serum, urine or exosomal samples and longitudinal follow-up.	Tissue profiling, serum/urine biomarkers, exosome analysis, clinical correlation and treatment-response modeling.	Prostate volume, IPSS, Qmax, PSA, inflammation/fibrosis scores, histological phenotype, progression and 5α-reductase inhibitor response; value requires clinical correlation or prediction (9, 49).

### 3.3 Hormonal signaling, inflammatory amplification, and tissue remodeling

Organelle stress-related abnormalities in BPH may have disease-specific features because prostate homeostasis is strongly influenced by hormonal signaling, metabolic status, and epithelial–stromal crosstalk (4, 51–53). Both prostate epithelial and stromal cells are regulated by androgen signaling. Enhanced DHT/AR signaling can promote cell proliferation and further amplify oxidative stress and inflammation through NOX4-related ROS production (54). AR signaling may also intersect with inflammatory pathways, including NF-κB and STAT3, resulting in mutual reinforcement among hormonal stimulation, inflammatory responses, and pro-survival signaling (55–57).

Beyond this indirect ROS-centered link, prostate-derived models provide more direct molecular clues connecting androgen signaling to MAM-associated machinery. In prostate cancer cells, androgen/AR activation has been shown to increase GRP78 expression, and DHT robustly upregulated GRP78 in LNCaP-derived models (58, 59). Given that GRP78 is independently reported to be upregulated in BPH tissue (7), this convergence provides a plausible androgen-sensitive bridge to MAM-associated stress regulation, although direct AR occupancy at the GRP78-encoding HSPA5 promoter and BPH-specific validation remain lacking. Sig-1R provides a second, more physical link: in prostate cancer models, Sig-1R physically associated with full-length AR and AR splice variants, whereas Sig-1R inhibition reduced DHT-induced AR nuclear translocation and AR stability (60). Importantly, that study did not establish that the Sig-1R–AR complex itself resides at MAMs. AR has also been detected in mitochondria of prostate cells and tissues and can influence oxidative phosphorylation and mitonuclear communication (61), further supporting organelle-proximal androgen signaling without proving MAM remodeling. We therefore propose two testable routes in BPH: a direct molecular-interface route involving androgen-sensitive GRP78 and Sig-1R–AR interaction, and an indirect route in which DHT/AR-driven NOX4/ROS and inflammatory stress could alter ER–mitochondria coupling. Whether such contact-site remodeling occurs in BPH remains to be established. At present, direct AR-dependent regulation of MFN2 or other MAM tethers in BPH remains unproven.

The pathological basis of BPH is not limited to epithelial proliferation. Rather, BPH reflects a chronic tissue remodeling process involving epithelial hyperplasia, stromal remodeling, an inflammatory microenvironment, and abnormal prostate smooth muscle function (62, 63). TGF-β–Smad signaling can promote EMT, stromal fibrosis, and extracellular matrix deposition (42). Inflammation-related mediators, including COX-2, PGE2, ADAM-17, EGFR, and ERK, may further connect chronic inflammation with prostate cell proliferation (64). Within this tissue context, organelle dysfunction may extend beyond single-cell stress responses and contribute to nodule formation, fibrosis progression, and heterogeneity in treatment response by altering epithelial–stromal signaling, inflammatory mediator release, metabolic adaptation, and thresholds for cell death.

From the perspective of organelle communication, hormonal signaling, the inflammatory microenvironment, ER stress, and mitochondrial injury in BPH should not be viewed as independent pathological events. MAMs may provide a candidate organelle-communication framework linking these processes. Whether ER–mitochondria contacts are structurally remodeled in BPH, and whether such changes contribute to disease progression, remain unproven (**Figure 2**). However, current evidence is still based mainly on indirect associations and mechanistic inference. Further studies using BPH tissues and relevant cellular models are required to validate structural alterations in MAMs, remodeling of key contact-site proteins, and their causal relationships with pathological phenotypes.

**Figure 2:**
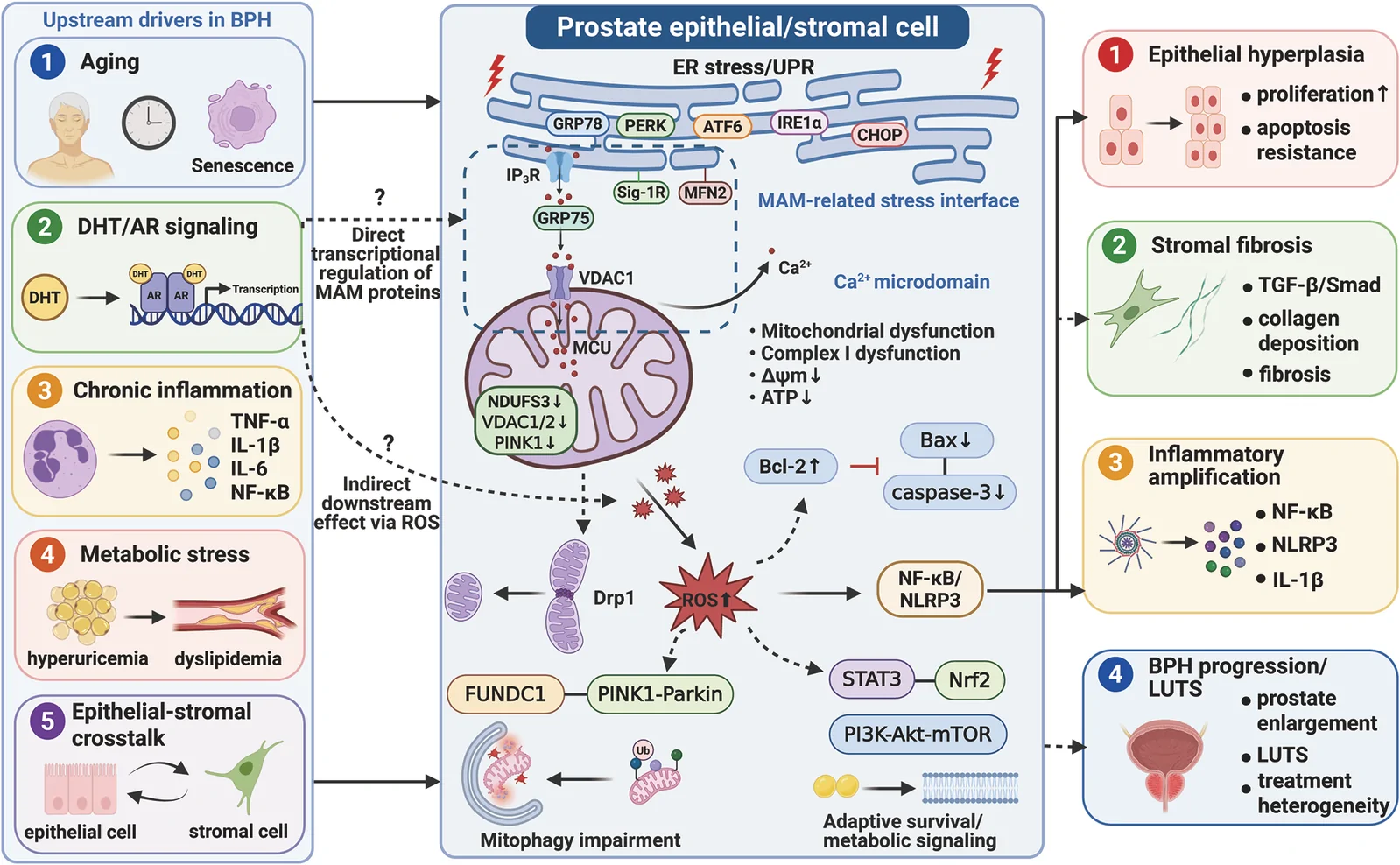
Schematic representation of core signaling mechanisms mediated by ER–mitochondria contact sites (MAMs) in prostate organelle communication. BPH-related upstream drivers, including ageing, enhanced DHT/AR signaling, chronic inflammation, epithelial–stromal crosstalk, and metabolic abnormalities, may affect mitochondrial function and quality control through ER stress, disturbed Ca²^+^ transfer, and ROS accumulation. For DHT/AR signaling, dashed arrows and question marks distinguish two hypothetical routes: possible direct transcriptional regulation of MAM-associated proteins and an indirect downstream effect mediated by ROS. Neither route should currently be interpreted as an established DHT/AR–MAM mechanism in BPH. Within this signal-integration framework, MAM-associated processes are linked to DRP1-dependent mitochondrial fission, PINK1–Parkin- and FUNDC1-related mitophagy, Bcl-2 family-mediated anti-apoptotic signaling, and ROS-associated inflammatory pathways such as NF-κB/NLRP3. These changes may contribute to epithelial proliferation, apoptosis resistance, stromal fibrosis, inflammatory amplification, and the broader pathological phenotype of BPH. Arrows and symbols indicate proposed activating or inhibitory relationships. By integrating Ca²^+^ homeostasis, ER stress, mitochondrial dynamics, autophagy, and inflammation, this schematic outlines a testable framework for investigating organelle communication in BPH.

## 4 Mechanistic hypotheses for the involvement of MAMs in BPH progression

### 4.1 Ca²^+^ imbalance and ROS amplification

On the basis of the functional properties of MAMs and the mitochondrial injury, oxidative stress, and inflammatory alterations observed in BPH, disturbed Ca²^+^ homeostasis and ROS amplification may represent central events in a MAM-related mechanistic model. The general bioenergetic consequences of altered MAM-mediated Ca²^+^ transfer are described in Section 2.2.1. Here, the BPH-specific question is whether disease-associated remodeling of ER–mitochondria contacts is sufficient to disturb mitochondrial bioenergetics and enhance ROS generation, as suggested by mechanistic studies in other systems (65–68). In BPH tissues, relatively consistent indirect evidence supports the presence of mitochondrial dysfunction and increased oxidative stress. Findings from both animal models and clinical samples suggest that hyperplastic prostate tissues may show ROS accumulation, reduced antioxidant capacity, and increased lipid peroxidation (41, 47). Multi-omics analyses further indicate that oxidative stress-related genes tend to be upregulated in the BPH microenvironment and are associated with increased cell proliferation and reduced apoptosis (69). Reduced expression of key mitochondrial respiratory chain proteins and impaired complex I function have also been linked to tissue fibrosis and functional abnormalities (49).

These findings support testing whether MAM-related Ca²^+^ transfer is altered in BPH and, if so, whether such alterations contribute to the coexistence of metabolic restriction and increased oxidative burden. This oxidative output may also connect organelle stress with innate immune activation. During NLRP3 inflammasome activation, NLRP3 and ASC have been reported to redistribute toward ER–mitochondria-associated regions, while ER–mitochondria communication can modulate inflammasome activity under cellular stress (12, 70). Inflammasome assembly promotes caspase-1 activation and the maturation of IL-1β and IL-18, providing a mechanism through which mitochondrial stress can be translated into inflammatory signaling. In BPH models, mitochondrial ROS accumulation and activation of the NLRP3/ASC/caspase-1/IL-1β pathway have already been reported (9, 71). Together, these observations support a testable mtROS–NLRP3–IL-1β/IL-18 axis in which MAMs may function as an immunometabolic interface between organelle stress and inflammatory amplification. However, direct MAM dependence of this axis has not yet been demonstrated in BPH.

### 4.2 ER stress adaptation and mitochondrial crosstalk

Several studies indicate that ER stress and UPR activation are present in BPH tissues. Alterations in GRP78, CHOP, and PERK-related pathways suggest that prostate cells may remain in a chronic stress state (72). In contrast to acute or severe stress, which often favors cell death, BPH cells retain substantial proliferative and survival capacity under chronic stress conditions, suggesting a stress-response pattern characterized by tolerance and adaptation. In this context, MAMs may function as a signal-integration platform connecting the UPR, mitochondrial metabolism, and cell fate regulation. PERK is not only an important UPR sensor but may also be enriched at ER–mitochondria contact sites, where it participates in stress-signal transmission between the ER and mitochondria (17, 32). Sig-1R and GRP78 contribute to protein-folding regulation and Ca²^+^ homeostasis at these sites and may influence both the magnitude and direction of stress-signal output. Stress-dependent remodeling of ER–mitochondria contacts may therefore influence mitochondrial adaptation and cell-fate signaling (14, 66).

In BPH tissues, long-standing inflammation and oxidative stress may continuously activate UPR pathways and relay stress signals to mitochondria through MAMs. Antioxidant and metabolic adaptation pathways, including Nrf2 and STAT3, may contribute to cellular tolerance to this unfavorable microenvironment (72–74). The UPR may also reduce apoptotic sensitivity by regulating Bcl-2 family proteins, further supporting cell survival (7). Thus, the MAM framework provides a testable mechanism through which persistent ER stress could be coupled to cellular adaptation, metabolic reprogramming, and proliferation-associated signaling.

### 4.3 Decline in mitochondrial quality control and ageing-related progression

BPH is strongly associated with ageing, and impaired mitochondrial quality control may provide an important link between ageing and prostate tissue remodeling. With advancing age, the balance between mitochondrial fission and fusion becomes more susceptible to disruption, mitophagy efficiency declines, and damaged mitochondria gradually accumulate. These damaged mitochondria may continuously release ROS, sustain low-grade chronic inflammation, and potentially promote hyperplastic remodeling (49, 75, 76).

Cellular senescence may provide an additional link between ageing and MAM-related stress in BPH. In non-prostatic models, increased ER–mitochondria contacts and ITPR2–MCU-dependent Ca²^+^ transfer promote mitochondrial Ca²^+^ accumulation, ROS production, and cellular senescence; enforced ER–mitochondria coupling can also induce a partly NF-κB-dependent senescence-associated secretory phenotype (SASP) (77). In the prostate, senescent epithelial and stromal cells can produce SASP factors, including IL-1α, IL-6, IL-8, and TNF-α, and can promote an inflammatory microenvironment and neighboring prostate-cell proliferation (78, 79). These observations raise the hypothesis that, if MAM remodeling occurs in BPH, it could connect organelle stress with senescence and SASP-driven epithelial–stromal crosstalk. However, this MAM–senescence–SASP axis has not been directly demonstrated in BPH and should remain a testable hypothesis. The proposed MAM-centered mechanisms linking organelle stress to BPH progression are summarized in **Figure 3**.

**Figure 3:**
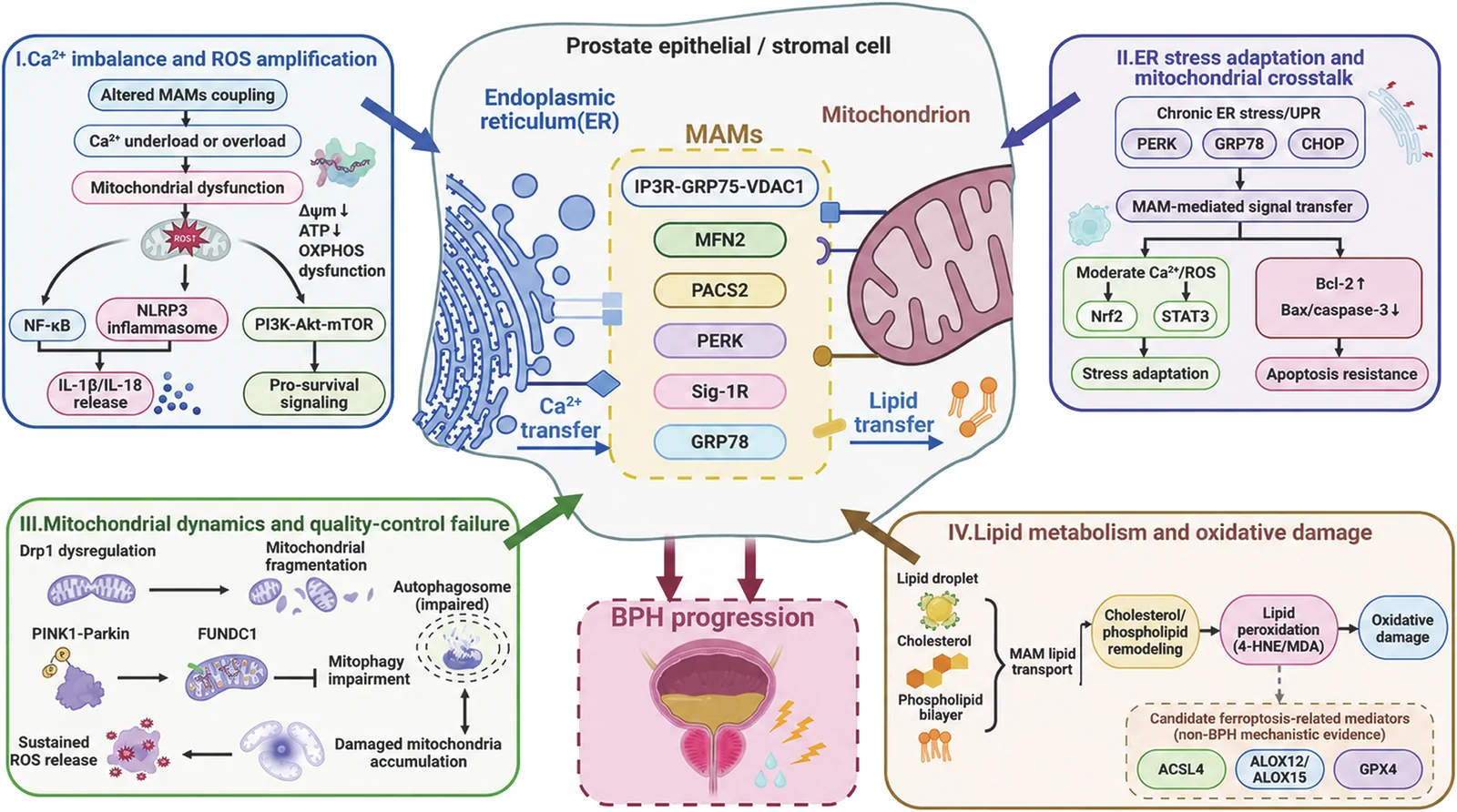
Proposed MAM-centered mechanisms linking organelle stress to BPH progression. The schematic integrates four interrelated modules: (I) Ca²^+^ imbalance and ROS amplification; (II) ER stress adaptation and mitochondrial crosstalk; (III) mitochondrial dynamics and quality-control failure; and (IV) lipid metabolism and oxidative damage. In Panel IV, candidate MAM-associated lipid-transfer proteins, including ORP5/ORP8, VPS13D, and ATAD3, are positioned upstream of cholesterol and phospholipid remodeling. Lipid peroxidation and the accumulation of products such as 4-HNE and MDA are shown as potential contributors to oxidative damage. ACSL4, ALOX12/ALOX15, and GPX4 are retained in a separate dashed annotation as candidate ferroptosis-related mediators supported by mechanistic studies outside BPH. They are not presented as an established MAM-dependent ferroptosis pathway in BPH.

MAMs provide a key spatial interface for mitochondrial dynamics and quality control. Mitochondrial fission often begins at local sites where the ER wraps around mitochondria and depends on the recruitment and activation of DRP1. The recognition, labeling, and clearance of damaged mitochondria involve pathways such as PINK1–Parkin and FUNDC1, whose effective operation also depends on the structural integrity and signaling efficiency of ER–mitochondria contact regions (27, 28, 30). When contact-site function is impaired, fission-site positioning may become abnormal, autophagy initiation may be weakened, and mitochondrial clearance may be delayed, ultimately reducing the efficiency of damaged mitochondrial removal.

In BPH research, reduced mitochondrial respiratory function and decreased expression of related proteins have been associated with ageing and tissue remodeling (49, 80). From the MAM perspective, these abnormalities may be interpreted as potential consequences of persistent damaged-mitochondria accumulation, manifested by sustained oxidative pressure, continued inflammatory signaling, and an increased tendency toward abnormal proliferation. Therefore, a potential MAM-related contribution to impaired mitochondrial quality control should be regarded as a candidate mechanism for chronic injury accumulation, persistent inflammation, and tissue remodeling in BPH, rather than as a confirmed pathogenic process.

## 5 Current evidence linking MAMs to BPH

### 5.1 Strength and limitations of current evidence

Current evidence indicates that MAMs may be associated with ER stress, mitochondrial dysfunction, increased oxidative stress, inflammatory amplification, and altered cell-death regulation in BPH. However, the MAM–BPH relationship remains largely at the level of mechanistic integration and hypothesis generation, with limited direct causal validation. To assess the strength of current support while avoiding direct extrapolation from other diseases to BPH, we distinguish direct evidence, strong indirect evidence, disease-context evidence, exploratory evidence, and translational evidence, together with their major limitations and priorities for future validation (**Table 3**). Overall, current support for MAM involvement in BPH is derived mainly from strong indirect evidence.

**Table 3:** Current evidence, major limitations, and research priorities for evaluating MAM involvement in BPH pathophysiology.

Evidence category	Current evidence base in BPH	Major limitation and research priority
Direct evidence, currently limited	Direct ER–mitochondria contact quantification, MAM protein localization and MAM-dependent flux data in BPH remain insufficient.	Prioritize TEM/3D-EM, super-resolution imaging, PLA/IF colocalization and contact-distance metrics in BPH tissues/models.
Strong indirect evidence: oxidative stress and inflammation	BPH repeatedly shows ROS accumulation, lipid peroxidation, antioxidant decline, cytokines, NF-κB and NLRP3 signaling (9, 71, 108).	Test whether MAM-node modulation changes mtROS, NF-κB, NLRP3, IL-1β and IL-18 in prostate models.
Strong indirect evidence: ER stress and UPR	GRP78 upregulation and PERK/ATF6/CHOP-related ER-stress responses are implicated in BPH (7, 46).	Combine ER-stress induction with MAM perturbation; measure GRP78, p-PERK, CHOP, mitochondrial function, ROS, proliferation and apoptosis.
Strong indirect evidence: mitochondrial dysfunction and mitophagy impairment	Respiratory dysfunction, damaged mitochondria, altered dynamics and impaired quality control are reported in ageing/stress-related BPH contexts (8, 49, 50).	Use mitophagy-flux assays with DRP1, PINK1/Parkin, FUNDC1, LC3, p62 and mitochondrial-clearance readouts.
Disease-context evidence	BPH involves ageing, AR signaling, chronic inflammation, metabolic stress, epithelial–stromal crosstalk, fibrosis and heterogeneity (109–111).	Correlate MAM indices with prostate volume, IPSS, Qmax, PSA, inflammation/fibrosis scores and 5α-reductase inhibitor response.
Exploratory evidence: lipid metabolism and ferroptosis-related susceptibility	Lipid metabolic disturbance, lipid peroxidation and ferroptosis-related susceptibility are suggested or inferred in BPH contexts (41, 112).	Treat as hypothesis-generating; combine lipidomics, lipid ROS, 4-HNE/MDA, GPX4/ACSL4 and ferroptosis intervention with MAM assays.
Translational evidence, not yet established	Oxidative/inflammatory markers may correlate with volume, PSA, symptoms or progression, but MAM-specific markers are not validated (109, 113, 114).	Build prospective cohorts integrating tissue MAM markers, serum/urine/exosomal biomarkers, histology, symptoms, flow parameters and follow-up.

From an evidence-hierarchy perspective, current support for MAM-related research in BPH is derived mainly from strong indirect evidence. The most relevant clues involve oxidative stress, chronic inflammation, ER stress and the UPR, mitochondrial dysfunction, and impaired mitochondrial quality control. Studies have reported ROS accumulation, enhanced inflammatory signaling, increased GRP78 and PERK-related stress responses, mitochondrial functional impairment, and a strengthened anti-apoptotic state in BPH tissues and models. These pathological alterations overlap substantially with core MAM functions, including Ca²^+^ transfer, ROS generation, UPR signaling, mitochondrial dynamics, and cell-fate regulation. The convergence of these processes suggests that MAMs may influence BPH-related phenotypes through the regulation of Ca²^+^ homeostasis and mitochondrial quality control. Nevertheless, functional overlap alone is insufficient to establish a direct role for MAMs in the initiation or progression of BPH. The weakest links in the current evidence chain include direct quantification of ER–mitochondria contact structures in BPH tissues, spatial mapping of MAM-associated proteins, dynamic assessment of MAM-dependent Ca²^+^ and ROS fluxes together with mitochondrial function, and phenotypic validation after intervention at key regulatory nodes. Based on the available evidence, MAMs may provide a potential site of convergence for several pathological processes in BPH, but they should not yet be regarded as established direct pathogenic factors.

### 5.2 Key questions and research gaps

Although several molecular clues have emerged, a complete evidence chain extending from structural quantification to clinical association is still lacking. At the structural level, direct evidence demonstrating MAM remodeling in BPH tissues is currently lacking. Systematic quantitative analyses of ER–mitochondria contact length, contact proportion, and contact frequency using transmission electron microscopy, three-dimensional reconstruction, or high-resolution imaging have not yet been systematically reported in BPH. As a complementary dynamic approach, genetically encoded split-GFP-based contact-site sensors such as SPLICS could be applied in living prostate cells and organoids to monitor ER–mitochondria proximity and distinguish narrow from wider contact populations under androgenic, inflammatory, or oxidative stress (81). Such studies would help determine whether MAMs in BPH are characterized by increased contact, reduced contact, or spatial reorganization, and may also distinguish organelle-communication features across different pathological subtypes.

At the molecular and cell-type levels, the expression profiles and spatial distribution of key MAM-associated proteins remain insufficiently defined. Existing studies suggest that HSP70 family members, such as GRP78 and HSPA1A, are dysregulated in hyperplastic prostate tissues or BPH models and are involved in cell proliferation, apoptosis regulation, oxidative stress, and stress adaptation. However, whether GRP75 is upregulated in BPH and contributes to IP3R–GRP75–VDAC1 complex-mediated ER–mitochondria crosstalk remains directly unverified (7, 82). Systematic single-cell and spatially resolved analyses are also lacking for MAM-associated molecules, including MFN2, VDAC1, PACS2, FUNDC1, Sig-1R, and PERK, across prostate epithelial, stromal, and immune cells. Future studies should therefore integrate single-cell and spatial approaches to define cell-type-specific patterns of MAM-associated molecules in prostate tissues.

A more critical limitation is the absence of causal evidence linking MAM alterations to BPH phenotypes. Future studies should directly manipulate key nodes, such as MFN2, GRP75, PERK, FUNDC1, DRP1, and Sig-1R, in BPH-1 cells, WPMY-1 cells, primary prostate cells, organoids, and animal models, followed by assessment of cell proliferation, apoptosis, inflammatory responses, mitochondrial quality control, and tissue remodeling. For *in vivo* causal testing, *Mfn2*fl/fl mice could be combined with a prostate epithelial Cre driver, such as PB-Cre4, to generate prostate epithelium-specific MFN2 loss of function. Because MFN2 has been reported to function either as an ER–mitochondria tether or as a spacer/negative regulator, the direction of the contact phenotype should not be prespecified (18, 83). Such experiments should determine whether MFN2 loss increases, decreases, or spatially redistributes ER–mitochondria contacts and whether these changes are accompanied by altered Ca²^+^ transfer and hyperplastic or tissue-remodeling phenotypes. Because MFN2 also regulates mitochondrial fusion, orthogonal contact-site measurements and rescue experiments would be required to distinguish MAM-dependent effects from broader mitochondrial-dynamics phenotypes. Only with this level of validation can MAMs move beyond mechanistic association and be evaluated as testable disease-regulatory nodes in BPH.

From a translational perspective, the association between MAM alterations and clinical phenotypes of BPH has not yet been established. Existing studies have shown that HSPA1A expression is positively correlated with prostate volume, total PSA, free PSA, and IPSS in patients with BPH. Multi-omics analyses and functional experiments further suggest that ACOX2 may be a candidate molecule in the oxidative stress microenvironment of BPH and may be associated with stromal cell proliferation and apoptosis resistance (69, 82). However, these indicators have not yet been incorporated into a MAM-related research framework. Future studies should examine whether MAM-associated molecular or structural alterations are related to prostate volume growth, inflammatory burden, histological phenotype, response to 5α-reductase inhibitors, and risk of disease progression. Linking organelle-communication changes with clinical phenotypes may help move MAM research from a basic mechanistic concept toward a potential entry point for BPH stratification and intervention-oriented studies.

## 6 Research prospects for BPH from the perspective of MAMs

### 6.1 Candidate molecules and mechanistic stratification

Current evidence on BPH indicates that disease heterogeneity extends beyond prostate volume and symptom severity to include inflammatory infiltration, oxidative stress burden, stromal remodeling, and differences among cellular subpopulations (75, 84, 85). This pathological diversity provides a rational basis for exploring MAM-associated molecules as markers for mechanistic stratification. MFN2, GRP75, VDAC1, GRP78, FUNDC1, and PERK are involved in organelle tethering, Ca²^+^ transfer, mitochondrial quality control, and ER stress responses. These molecules may therefore serve as candidate indicators of cellular stress burden, metabolic status, and inflammatory signaling activity.

The value of these molecules should not be framed primarily as diagnostic markers for BPH. They may be more useful as auxiliary indicators for interpreting interpatient differences in disease mechanisms. Future studies should examine their associations with prostate volume, IPSS score, maximum urinary flow rate, response to 5α-reductase inhibitors, and risk of disease progression. Study designs should also distinguish among three levels of evidence: changes in the expression of MAM-associated proteins, structural alterations in ER–mitochondria contacts, and measurable changes in functional flux. This distinction is important because altered expression of a single molecule cannot be taken as sufficient evidence of MAM dysfunction.

### 6.2 Potential intervention pathways and verifiable targets

At present, α1-adrenoceptor blockers and 5α-reductase inhibitors remain the pharmacological cornerstones of BPH treatment. α1-Adrenoceptor blockers mainly relieve voiding symptoms related to smooth muscle tone, whereas 5α-reductase inhibitors slow prostate enlargement by suppressing androgen metabolism (86–88). Beyond these established clinical effects, whether standard BPH therapies alter ER–mitochondria communication remains poorly defined. Finasteride provides an important example because available studies suggest effects on both ER-stress and mitochondrial pathways.

In a testosterone-induced BPH model, finasteride reduced oxidative and inflammatory responses and suppressed ER-stress-associated changes involving GRP78, CHOP, PERK–eIF2α, and IRE1α–XBP1 signaling (45). These effects indicate that finasteride can modify ER-stress pathways that are functionally connected to MAM biology. Human prostate tissue data provide a complementary mitochondrial observation. Short-term exposure to finasteride, alone or with celecoxib, was associated with reduced NDUFS3 expression, suggesting an effect on mitochondrial Complex I-related bioenergetic status (80). Clinically, finasteride reduces prostate volume and can improve LUTS-related outcomes, although the magnitude and time course of symptom improvement differ from those of α1-adrenoceptor blockade or combination therapy (88). Taken together, these findings suggest that finasteride may influence both ER and mitochondrial homeostasis. However, none of these studies directly assessed ER–mitochondria contact structure, MAM-associated protein proximity, or MAM-dependent functional flux. Therefore, a direct effect of finasteride on MAM remodeling has not been established. Future studies combining treatment-response assessment with MAM structural and functional measurements will be required to determine whether 5α-reductase inhibition alters ER–mitochondria coupling in BPH.

Based on the available evidence, indirect modulation of MAM-related pathological processes appears to be a more realistic near-term direction. Several animal and cellular studies of BPH have shown that interventions targeting oxidative stress, inflammation, and cell survival pathways can improve hyperplasia-related phenotypes (9, 89, 90). These findings do not demonstrate that such effects are MAM-dependent. Nevertheless, they suggest that oxidative stress, inflammatory responses, and pro-survival signaling may participate in phenotypic regulation, potentially through changes in ER–mitochondria functional coupling.

Improving mitochondrial function is another indirect intervention pathway that deserves attention. Studies of age-related BPH have shown that mitochondrial complex I dysfunction is associated with prostate fibrosis and voiding dysfunction, whereas oleic acid supplementation can partially restore mitochondrial function and alleviate related pathological alterations (49). This finding suggests that improving mitochondrial bioenergetic status may indirectly affect MAM-associated Ca²^+^ transfer, ROS generation, and organelle quality control. Metabolic regulatory nodes such as SIRT1, SIRT3, AMPK, and PGC-1α may also be considered in future studies, because these pathways are closely related to mitochondrial homeostasis, antioxidant defense, and cellular adaptation to stress (91, 92).

Compared with indirect interventions, direct regulation of MAM structure or tethering complexes remains exploratory. MFN2, Sig-1R, and the IP3R–GRP75–VDAC1 axis have been widely studied in neurodegenerative, metabolic, and cardiovascular diseases, but their roles in BPH have not been systematically validated. At present, these molecules should therefore be regarded as mechanistic research nodes rather than mature therapeutic targets for BPH.

### 6.3 Experimental models and validation strategies

To move the MAM–BPH hypothesis from mechanistic interpretation toward experimental validation, future studies should combine human tissues, cellular models, organoids, animal models, and clinical cohorts. Rather than measuring individual MAM-associated molecules alone, these studies should assess ER–mitochondria contact structures, the spatial localization of key proteins, functional fluxes, and their relationships with BPH phenotypes.

Cellular models and co-culture systems are suitable for dissecting the effects of candidate nodes, including MFN2, GRP75, PERK, FUNDC1, DRP1, and Sig-1R, on Ca²^+^ homeostasis, ROS generation, mitochondrial function, ER stress, inflammatory responses, and the balance between proliferation and apoptosis. Some of these nodes are currently supported mainly by basic studies of MAM biology and still require validation in BPH-specific models (9, 83, 93). Organoid and tissue-engineered models may help bridge cellular mechanisms with patient-derived sample heterogeneity, whereas animal models are useful for evaluating system-level associations among MAM alterations, prostate weight, tissue remodeling, and voiding function (49, 94–96).

**Table 2** summarizes complementary experimental models, methodological approaches, and key criteria for evaluating the MAM–BPH hypothesis. These approaches do not assume that MAMs are established pathogenic factors in BPH. Instead, they provide practical options for progressing from mechanistic hypotheses toward causal validation.

## 7 Conclusion

BPH should be understood not only as a morphological enlargement of the prostate but also as a chronic, ageing-related progressive disease. Its pathological evolution involves chronic inflammation, remodeling of hormonal signaling, changes in the tissue microenvironment, and disruption of organelle homeostasis. Available evidence indicates that BPH is frequently accompanied by ER stress, mitochondrial dysfunction, oxidative stress, altered mitochondrial dynamics, inflammatory amplification, and resistance to cell death. These abnormalities are unlikely to occur as entirely independent events; rather, they may converge at ER–mitochondria contact sites, forming an interactive regulatory network that links ER stress, mitochondrial injury, oxidative stress, and inflammatory signaling during disease progression.

MAMs regulate Ca²^+^ transfer, lipid metabolism, mitochondrial bioenergetics, mitochondrial dynamics, autophagy, and inflammatory signal integration. This functional range makes them a useful organelle-communication framework for interpreting the multiple pathological phenotypes observed in BPH. In the context of persistent low-grade inflammation, metabolic stress, and ageing-related loss of homeostatic control in the prostate, MAM dysfunction may affect stress adaptation, sustained proliferative activity, and tissue remodeling. However, direct evidence demonstrating MAM alterations in BPH remains limited. MAMs should therefore be considered a candidate mechanistic hub requiring further validation, rather than an established pathogenic factor.

Future studies should systematically assess the structural remodeling of MAMs, the expression and localization of key tethering proteins, their cell-type-specific distribution, and their functional consequences in BPH tissues and relevant experimental models. Integrated analyses using human prostate tissues, BPH cell models, organoids, and animal models may help determine whether MAM alterations contribute to disease initiation, progression, and heterogeneity in therapeutic response. If causal links are confirmed, MAM-focused research could refine current understanding of organelle pathology in BPH and open new directions for disease stratification, biomarker discovery, and targeted intervention.
